# Supplementary material for: Resolving the full spectrum of human genome variation using Linked-Reads
Source: Genome Res. 2019 Apr;29(4):635–45. doi: 10.1101/gr.234443.118 (PMC6442396; doi:10.1101/gr.234443.118)
Supplement: Supplemental Material [file supp_gr.234443.118_Supplemental_longranger-2.2.zip › longranger-2.2.tar.bz2!/lr2.2-public/lib/go/src/code.google.com/vitess/docs/resources/openworld-2015-vitess.pdf]

YouTube Vitess

Cloud-Native MySQL

Oracle OpenWorld Conference

October 26, 2015

Anthony Yeh, Software Engineer, YouTube

Spoiler Alert

Spoilers

1. History of Vitess
2. What is Cloud-Native Computing?
3. What is Cloud-Native MySQL?
4. Vitess Architecture
5. Transparent Live Resharding
6. Example Resharding Workflow
7. Cloud Scaling Benchmarks
8. Vitess Roadmap
9. It was all a dream

Histoir de Vitess

Vitess 1.0

golang.org

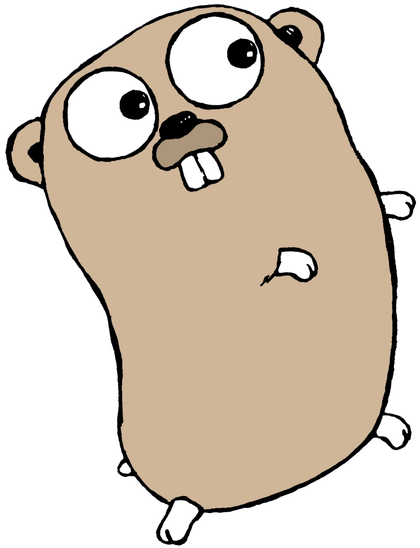

- YouTube is a MySQL app
- Vitess started in YouTube data centers
  - Connection-pooling proxy
- Early adopter of Go (golang.org)
  - First commit (in original repo) in 2010
  - 2 years before Go 1.0
  - Cheap connections, goroutines
- In production at YouTube since 2011

Vitess 1.5

- YouTube moved into Borg[1]
  - Google MySQL
- Adapted Vitess to Borg environment
  - Dynamically scheduled container cluster
- Over time, Vitess evolved within Borg
  - Database protection
  - Query rewriting/blacklisting
  - Row-based cache
  - Shard routing
  - Cluster management
  - Monitoring

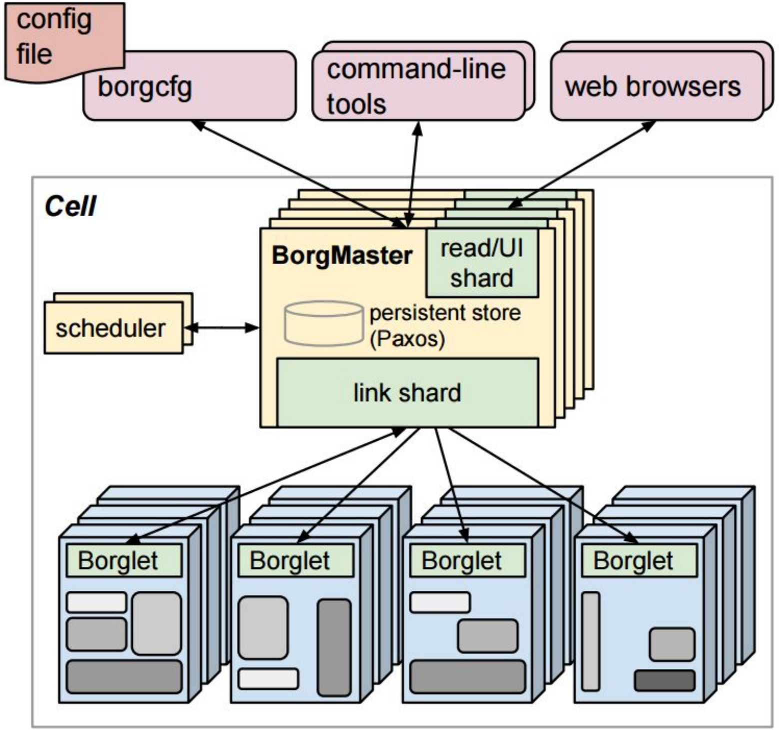

[1] http://research.google.com/pubs/pub43438.html

Growing with YouTube

- YouTube stats[1]
  - >1B users
  - 400 hrs/min of new videos (24K s/s)
  - 80% of views from outside U.S.
- Schema constantly evolving
  - Content ID, channel admin, live streams, video editing, music
- Developed live resharding

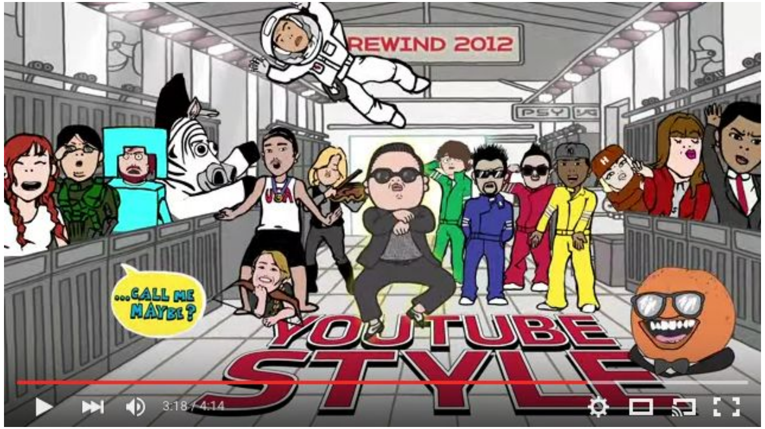

[1] youtube.com/yt/press/statistics.html

Vitess 2.0

Vitess in 2014

- Worked great in YouTube, but outside…
  - Users can't get past build step
  - Custom patched MySQL
  - No docs for setting up a deployment
- Along comes Kubernetes
  - Like open-source Borg

Vitess in 2015

- Bring-your-own MySQL 5.6
- Docker images
- Out-of-the-box deployment config
  - Runs on any cloud platform supported by Kubernetes (AWS, Azure, GCP, …)
- Lots more documentation
- Step-by-step guides

Cloud-Native Computing

Cloud Native Computing Foundation (cncf.io)

Distributed systems paradigm

- Container packaged
    - Dependency management
    - Resource isolation
- Dynamically scheduled
    - Increase utilization
    - Simplify operations
- Microservices-oriented
    - Distributed applications
    - Service endpoints

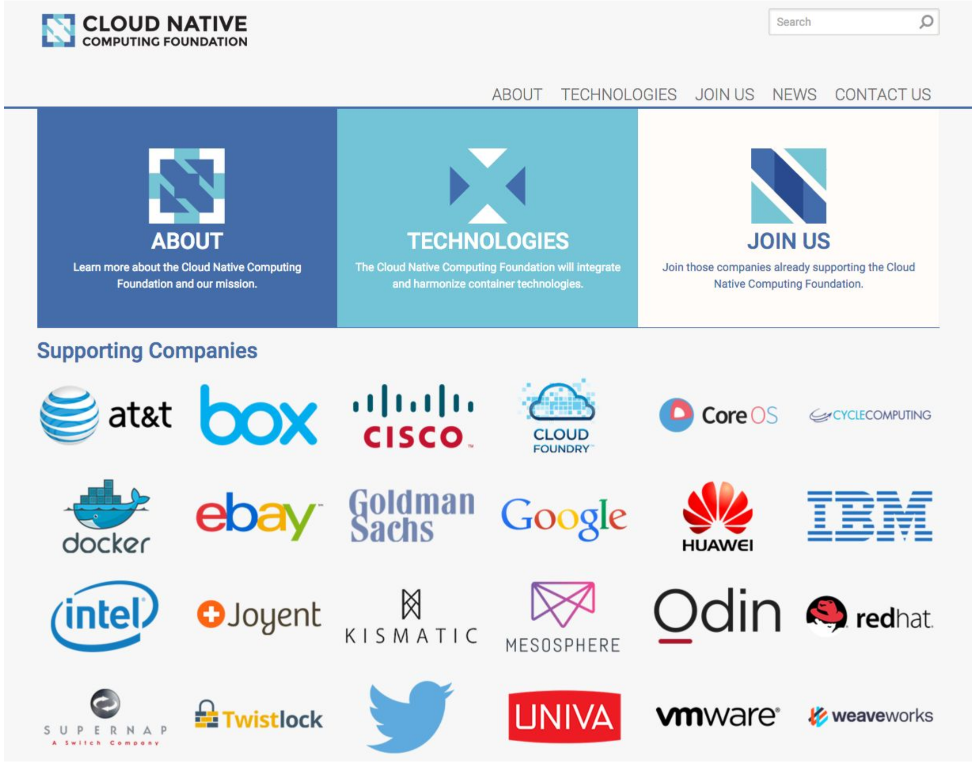

Kubernetes (κυβερνήτης)

kubernetes.io

- Provides glue for distributed apps
  - Dynamic scheduling
  - Declarative deployment config
  - Service discovery
  - High-availability replica pools
  - Rolling updates
  - Component grouping/metadata
- Abstracts cloud-platform-specific pieces
  - VM instance creation and config
  - Networking config
  - External load balancers
  - Persistent storage volumes (PD, EBS)

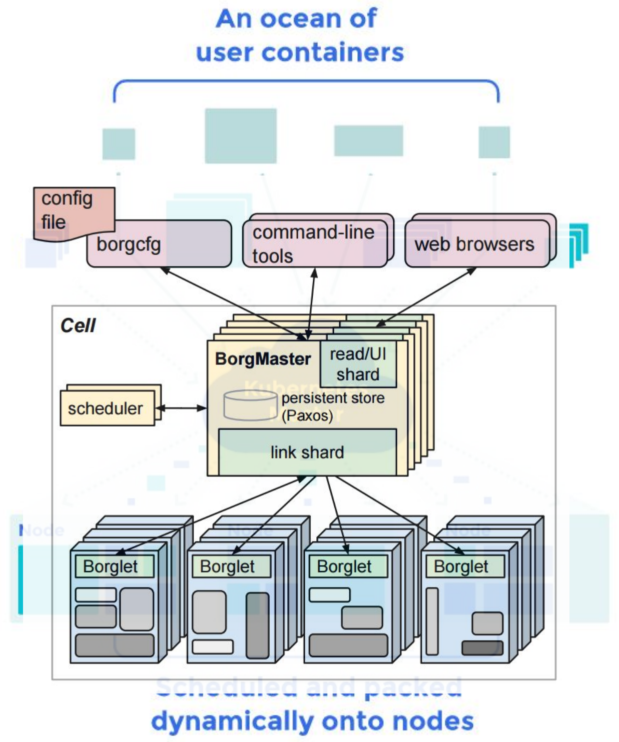

Cloud-Native MySQL

Pets vs. Cattle[1]

Your servers might be **pets** if...	Your servers might be **cattle** if...
You can log into them by hostname or IP.	You know only that a bunch of them are out there, somewhere.
You load data onto them and tell them what to do.	They go off and join the herd without being told to.
You try to fix them when they go down.	You wait for others to take over their jobs when they go down.
Your app knows which servers to send which queries to.	Your app throws queries over a magic wall and results appear.
You know when your last master failover was.	You have a monitoring graph of failovers per day.

[1] Noah Slater, blog.engineyard.com/2014/pets-vs-cattle

Dynamic Scale-Out

- Scale-out by adding more cattle to the herd

```
$ kubectl create -f vttablet.yaml
```

- Dynamic scheduling means you don't worry about...
  - What server is this going to be launched on?
  - How is it going to get a snapshot of existing data?
  - How is it going to find and connect to the master?
  - How are app servers going to find out it exists?
- With live resharding, it even works for scaling master traffic.

Query Routing as a Service

- Separation of concerns
  - App doesn't know anything about cluster topology
  - MySQL doesn't know it's inside Kubernetes
  - Vitess connects everything
- Distributed Vitess components
  - Use service discovery to find each other
  - Communicate over defined RPC APIs

Vitess Architecture

Vitess Components

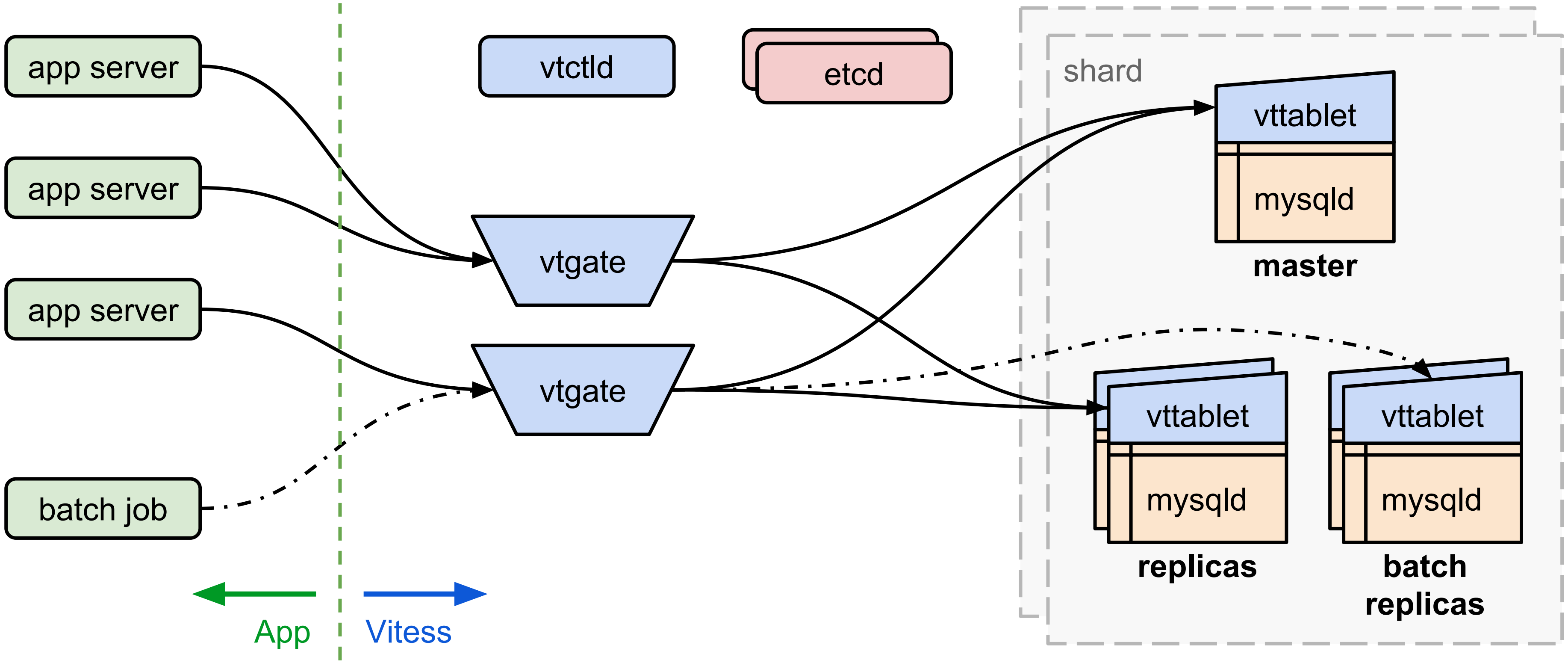

Vitess Concepts

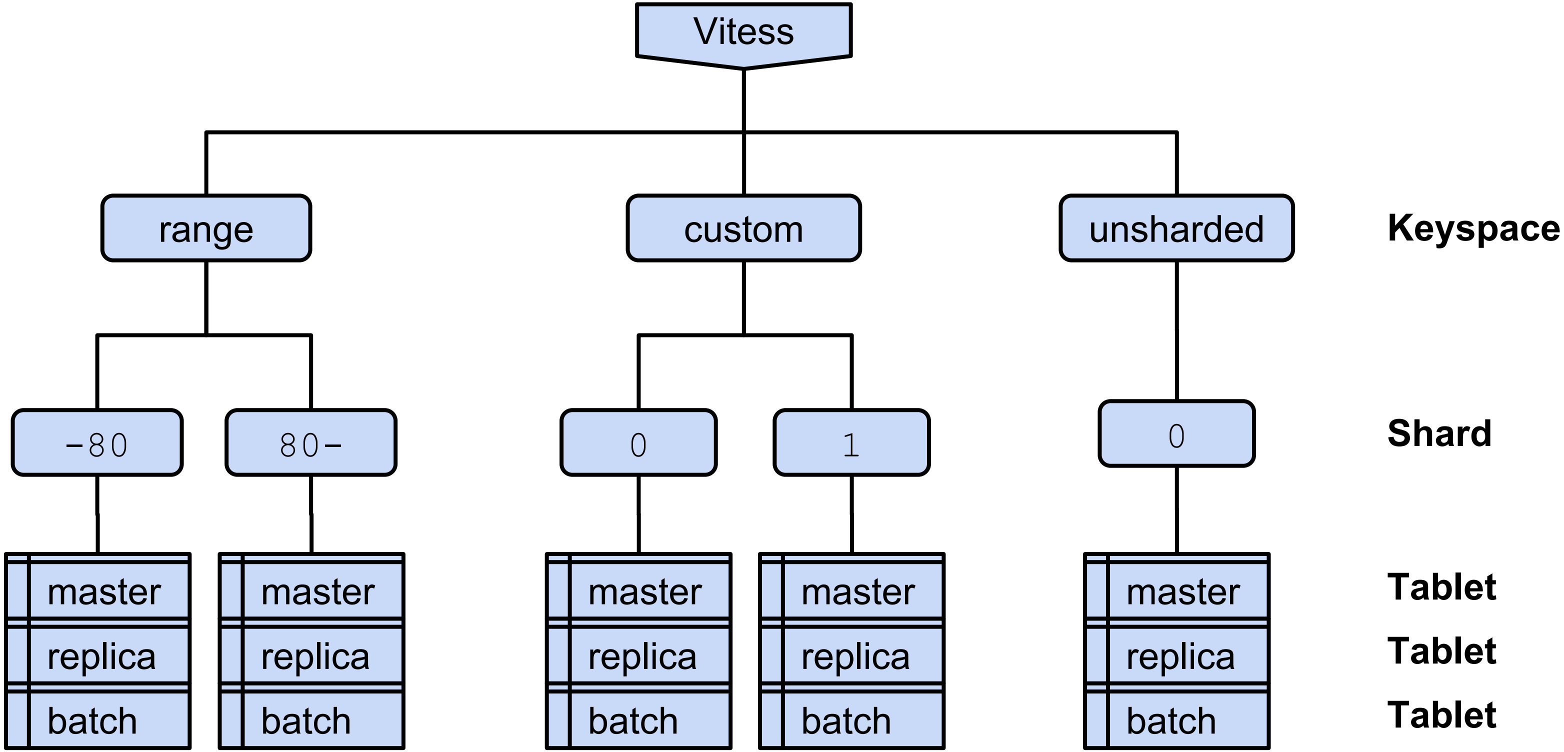

Range-Based Sharding

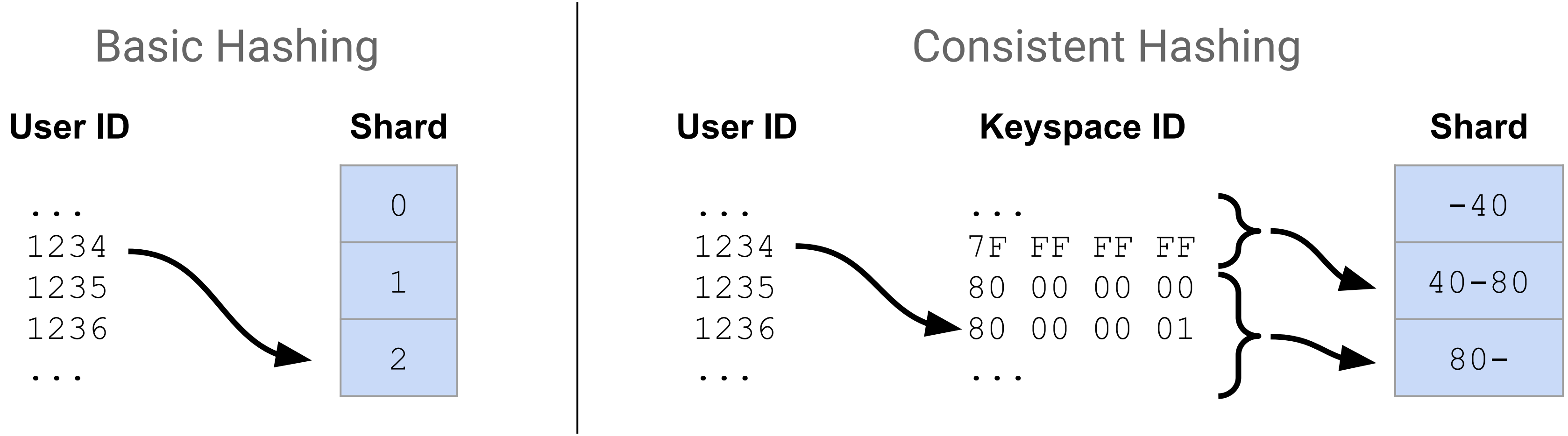


```
cursor = conn.cursor('test_keyspace', 'replica', keyspace_ids=[my_hash(user_id)])
cursor.execute(
  'SELECT message FROM messages WHERE user_id=%(user_id)s ORDER BY time_created_ns',
  {'user_id': user_id})
return [row[0] for row in cursor.fetchall()]
```

Transparent Live Resharding

Vitess Resharding

Before

User ID	Keyspace ID
...	...
1234	7F FF FF FF
1235	80 00 00 00
1236	80 00 00 01
...	...

Shard	Key Range
-40	00 00 00 00 - 3F FF FF FF
40-80	40 00 00 00 - 7F FF FF FF
**80-**	**80 00 00 00 - FF FF FF FF**

After

User ID	Keyspace ID
...	...
1234	7F FF FF FF
1235	80 00 00 00
1236	80 00 00 01
...	...

Shard	Key Range
-40	00 00 00 00 - 3F FF FF FF
40-80	40 00 00 00 - 7F FF FF FF
**80-C0**	**80 00 00 00 - BF FF FF FF**
**C0-**	**C0 00 00 00 - FF FF FF FF**

Transparent to the Application

- The app has no knowledge of what shards exist.
- The app keeps running normally during resharding.

```
def my_hash(user_id):
  m = hashlib.md5()
  m.update(uint64.pack(user_id))
  return m.digest()[:8]
```

```
cursor = conn.cursor('test_keyspace', 'replica', keyspace_ids=[my_hash(user_id)])
cursor.execute(
  'SELECT message FROM messages WHERE user_id=%(user_id)s ORDER BY time_created_ns',
  {'user_id': user_id})
return [row[0] for row in cursor.fetchall()]
```

Live Migration

- Old and new shards overlap during migration.

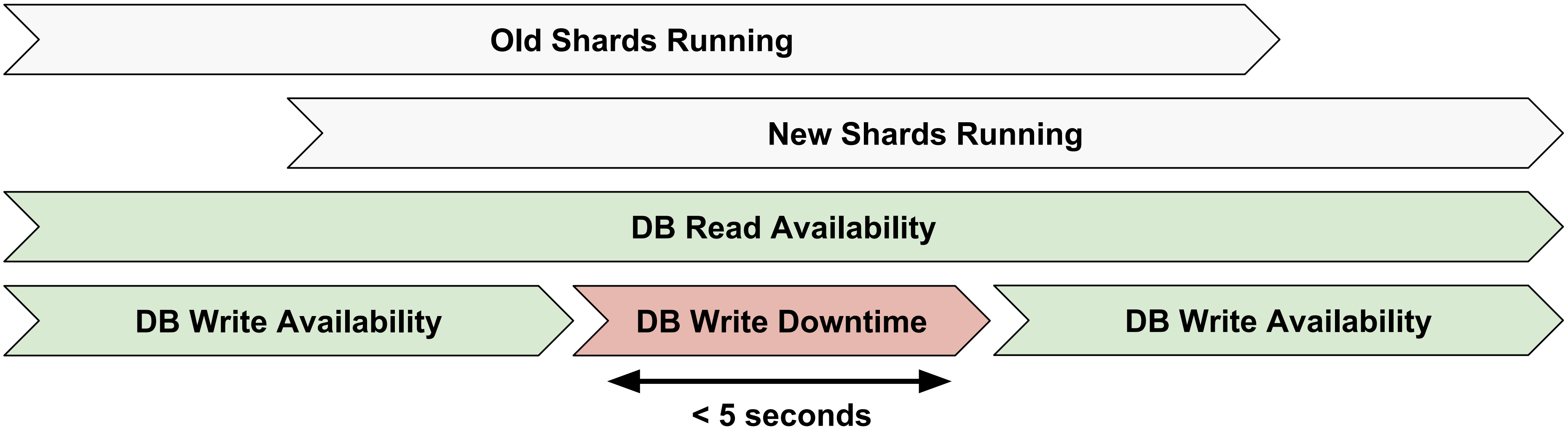

Masters are cattle too

- Transparent Live Resharding means you can scale master traffic too, by adding cattle.
- When resharding stops being scary…
  - You don't have to predict and provision for years of growth.
  - Provision for good utilization.
  - Start small and scale out as needed.
  - Cool down hot shards one at a time.

Resharding Workflow

vitess.io/user-guide/sharding-kubernetes.html

Prepare Schema

- Initial sharding
  - Add Keyspace ID column
  - Populate along with new rows
  - Backfill Keyspace ID for existing rows
- Example: Multi-tenant Guestbook (vitess.io/getting-started/)

```
CREATE TABLE messages (
  page BIGINT(20) UNSIGNED,
  time_created_ns BIGINT(20) UNSIGNED,
  keyspace_id BIGINT(20) UNSIGNED,
  message VARCHAR(10000),
  PRIMARY KEY (page, time_created_ns)
) ENGINE=InnoDB
```

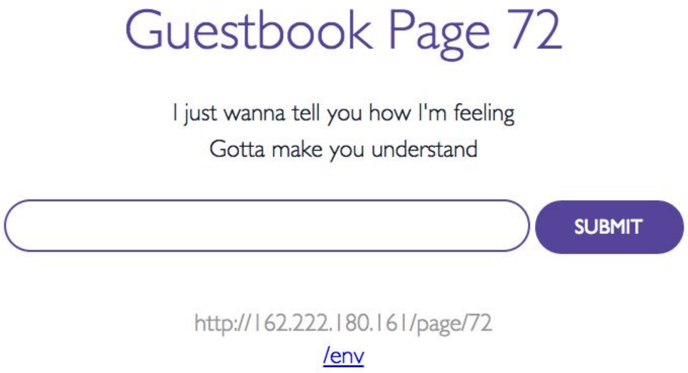

Provision New Shards

Moo

```
$ kubectl create -f vttablet.yaml
```

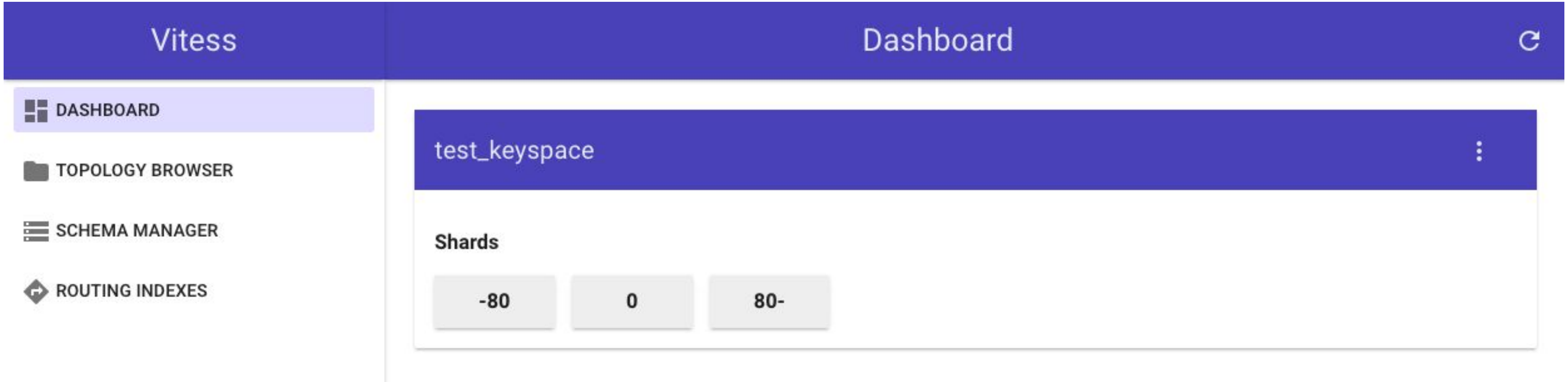

Copy Data

- Split Clone
  - Pauses replication on a backup slave
    - So it's consistent with a known GTID set
  - Streams rows to new shards
    - Doesn't use extra disk space on source shard

```
$ vtworker SplitClone --strategy=-populate_blp_checkpoint test_keyspace/0
```

Filtered Replication

- Vitess annotates DMLs with the Keyspace ID
- New shards subscribe to filtered replication stream
- Let new shards catch up to old shard master

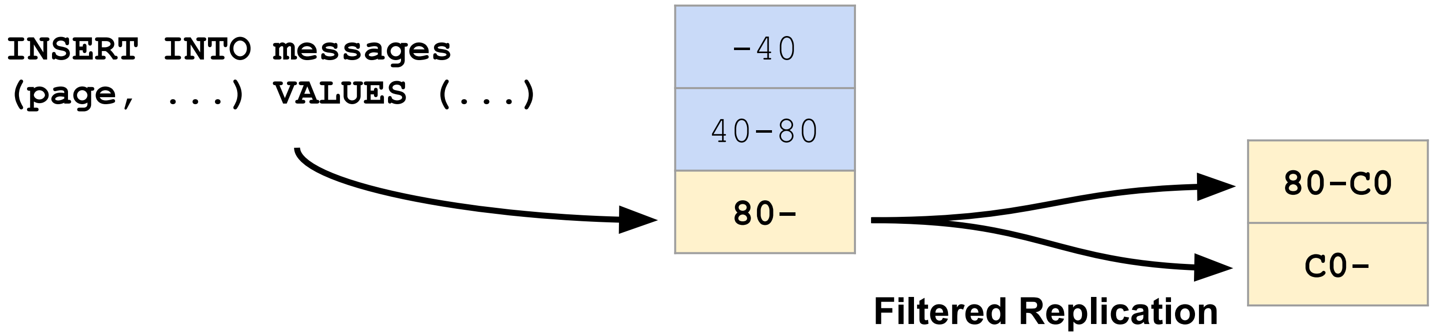

Verify Data Integrity

- Split Diff
  - Pauses replication on backup slave in old shard
  - Pauses filtered replication on new shards
    - At the equivalent GTID set
  - Performs a row-by-row comparison
    - Also detects rows only present on one side

```
$ vtworker SplitDiff test_keyspace/0
```

Shard Migration

Migrate non-master traffic to new shards

```
$ vtctlclient MigrateServedTypes test_keyspace/0 replica
```

Migrate master traffic to new shards

```
$ vtctlclient MigrateServedTypes test_keyspace/0 master
```

Scaling Benchmarks

Scaling Writes by adding Shards

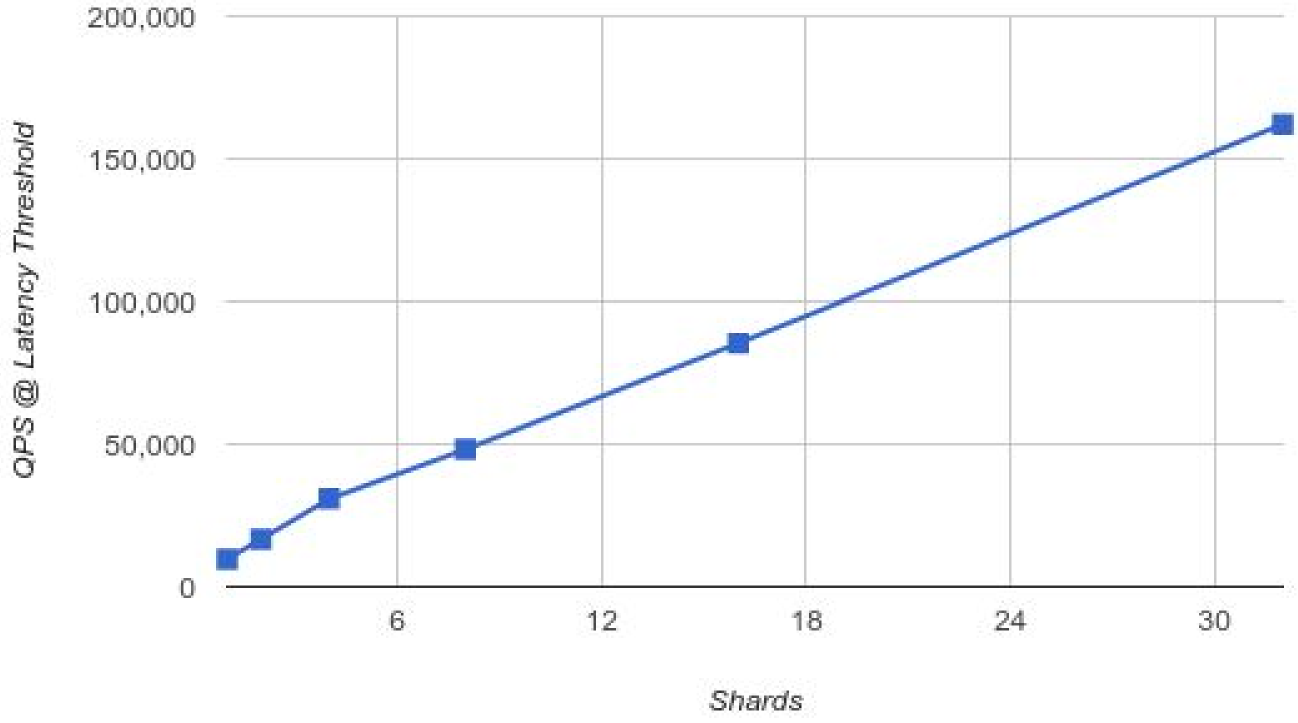

Scaling Reads by adding Replicas

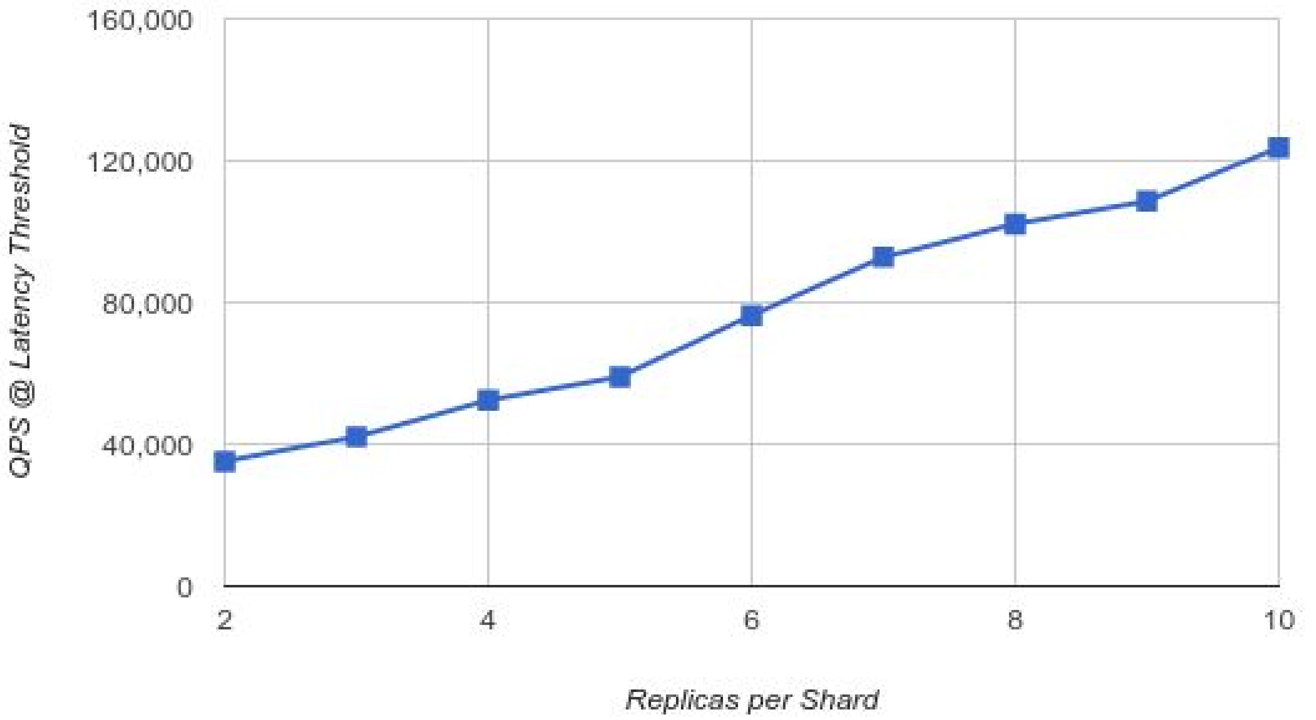

Vitess Roadmap

Vitess 2.0.0

- Stable releases
    - API freeze (no breaking changes)
    - Release changelogs (github.com/youtube/vitess/releases)
    - Versioned Docker images (hub.docker.com/u/vitess/)
- What's new in 2.0
    - Deployment configs for Kubernetes 1.0
        - Users reported success on both AWS and Google Cloud Platform
    - Official client libraries for Java, Python, PHP, Go, and Hadoop
    - MySQL 5.6 support
    - Built-in cloud backup/restore
    - HTTP/2-based gRPC protocol (grpc.io)

Future Plans

- Drop-in drivers for JDBC, PDO, PEP 249
    - Already have Go driver for database/sql
- Cross-shard joins/aggregations
- Automatic master election
- Automated out-of-band schema changes

Resources

Try Vitess

vitess.io/getting-started/

vitess.io/user-guide/sharding-kubernetes.html

Contribute

github.com/youtube/vitess

Contact Us

vitess@googlegroups.com

Get Updates

groups.google.com/d/forum/vitess-announce

Cloud Native Computing Foundation

cncf.io

Kubernetes

kubernetes.io
